# Assessment of modelling strategies for drug response prediction in cell lines and xenografts

**DOI:** 10.1038/s41598-020-59656-2

**Published:** 2020-02-18

**Authors:** Roman Kurilov, Benjamin Haibe-Kains, Benedikt Brors

**Affiliations:** 1 Division of Applied Bioinformatics, German Cancer Research Center, Heidelberg, Germany; 2 Faculty of Biosciences, Heidelberg University, Heidelberg, Germany; 3 Princess Margaret Cancer Centre, Toronto, Ontario M5G 1L7 Canada; 4 Department of Medical Biophysics, University of Toronto, Toronto, Ontario M5G 1L7 Canada; 5 Department of Computer Science, University of Toronto, Toronto, Ontario M5T 3A1 Canada; 6 Ontario Institute for Cancer Research, Toronto, Ontario M5G 1L7 Canada; 7 National Center for Tumor Diseases (NCT), Heidelberg, Germany; 8 German Cancer Consortium (DKTK), Core Center Heidelberg, Heidelberg, Germany

**Keywords:** Machine learning, Predictive medicine, Cancer

## Abstract

Data from several large high-throughput drug response screens have become available to the scientific community recently. Although many efforts have been made to use this information to predict drug sensitivity, our ability to accurately predict drug response based on genetic data remains limited. In order to systematically examine how different aspects of modelling affect the resulting prediction accuracy, we built a range of models for seven drugs (erlotinib, pacliatxel, lapatinib, PLX4720, sorafenib, nutlin-3 and nilotinib) using data from the largest available cell line and xenograft drug sensitivity screens. We found that the drug response metric, the choice of the molecular data type and the number of training samples have a substantial impact on prediction accuracy. We also compared the tasks of drug response prediction with tissue type prediction and found that, unlike for drug response, tissue type can be predicted with high accuracy. Furthermore, we assessed our ability to predict drug response in four xenograft cohorts (treated either with erlotinib, gemcitabine or paclitaxel) using models trained on cell line data. We could predict response in an erlotinib-treated cohort with a moderate accuracy (correlation ≈ 0.5), but were unable to correctly predict responses in cohorts treated with gemcitabine or paclitaxel.

## Introduction

Drug response prediction based on genomic information is an active area of research with many practical applications including drug discovery, drug repurposing, patient selection for clinical trials, and personalized treatment recommendations (e.g. in a tumorboard setting). Several large-scale cell line drug sensitivity screens have been generated by the scientific community (e.g. CCLE^1^, CTRP^2^, GDSC^3^, gCSI^4^). These datasets contain molecular and drug response data on hundreds of cell lines, allowing for generation of predictive models. But despite the availability of such training data, our ability to accurately predict drug response still remains quite limited^5,6^. Reasons that make drug response prediction a hard problem include noise in the data, relatively low number of samples compared to the number of features (i.e. predictor variables), incomplete omics characterization, and the static nature of molecular data. Molecular data in such studies are usually acquired only before drug treatment^7^. Another important problem is the consistency of pharmacogenomics associations derived from different datasets. A number of studies examined agreement between the largest datasets and found that differences in experimental protocols and differences in data analysis likely contributed to the observed inconsistency^8–13^.

A broad spectrum of machine learning methods has been applied to the drug response prediction problem: regularized regression methods (e.g. lasso, elastic net, ridge regression)^1,3,4,14–17^, partial least squares (PLS) regression^18^, support vector machines (SVM)^19^, random forest (RF)^3^, neural networks and deep learning^20,21^, logical models^3^, or kernelised bayesian matrix factorization (KBMF)^22,23^. For a comprehensive recent review see Ali & Aittokallio (2018)^24^. However, no systematic exploration of model training strategies based on data from multiple large cell line screens has been reported so far. Also cell line-based models have not yet been compared to xenograft-based models. In the current study we attempt to close these gaps with the ultimate goal of improving accuracy of drug response prediction in cell lines and xenografts.

For our analyses we used data from the cancer cell line encyclopedia^1^ (CCLE), cancer therapeutics response portal^2^ (CTRP), genomics of drug sensitivity in cancer^3^ (GDSC) and the genentech cell line screening initiative^4^ (gCSI), together with the only publicly available xenograft screen ‒ Novartis Institutes for BioMedical Research PDX Encyclopedia^25^, NIBR PDXE (Table 1).

**Table 1:** Datasets used in the study and corresponding sample sizes.

Dataset	# of cell lines	# of drugs
CCLE^1^	505	24
CTRP^2^	860	481
GDSC^3^	1000	265
gCSI^4^	410	16
NIBR PDXE^25^	23–38 xenografts per drug	62

All datasets have expression, copy number and mutation data, GDSC dataset also provides cell line methylation data.

**Figure 1:**
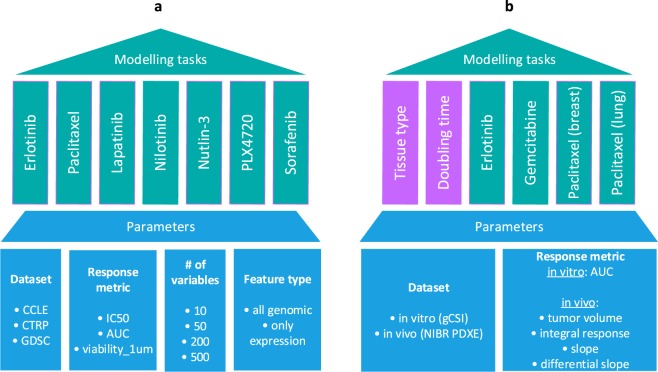
Experimental design of the study. The study is organized in two parts. (**a**) We examine models for seven different drugs using data from 3 large cell line datasets (CCLE, CTRP and GDSC) by varying feature type, response metric and number of features used. (**b**) We compare models for 3 different drugs (in 4 tissues) trained on cell line data (gCSI) with the matching models trained on xenograft data (NIBR PDXE); for xenografts we also compare 4 different drug response metrics. Additionally we compare drug response models with tissue type and doubling time models in terms of accuracy.

We first set out to systematically study the influence of different modelling parameters (e.g. response metric, number of features, type of features) on predictive performance in drug sensitivity testing. We included data from three large cell line-based datasets, CCLE, GDSC and CTRP, respectively (Fig. 1a). Only seven drugs were in overlap between these screens, and we predicted drug sensitivity for all of them in each dataset. Predictive performance was assessed by cross-validation. We then took the best parameter set and strategy from this analysis and checked whether drug sensitivity in animal xenograft systems could be predicted by training of models from cell line-based data (Fig. 1b). For this, we chose the gCSI and NIBR PDXE datasets, where only three drugs could be compared. Since in xenograft experiments each drug was tested on samples from a particular tissue type, we also restricted cell line samples accordingly, e.g. for erlotinib response prediction all xenograft samples belonged to the non-small-cell lung cancer type and all cell line samples used for this modelling task were lung cancer cell lines. We included, as a background, two additional variables to predict, tissue type and growth rate/doubling time, which should present comparatively easier prediction tasks.

## Data and Methods

### Data types and sources

For modelling we used molecular, drug response, tissue type, and division rate data from 4 large cell line sensitivity screenings ‒ CCLE^1^, CTRP^2^, GDSC^3^, gCSI^4^ and one xenograft screen ‒ NIBR PDXE^25^.

#### Cell line molecular data

Molecular data included gene expression, copy number information, and mutation information. Expression data comes from microarrays (except for gCSI dataset where it comes from RNA-seq), values are continuous. Copy number information comes from SNP arrays, it’s continuous. Mutation information is derived from whole exome sequencing and is represented by binary values per gene (1/0 for presence/absence of mutation).

Molecular information for CCLE, CTRP and GDSC datasets was obtained from the PharmacoGx package (version 1.8.3)^26^. Preprocessing details for each dataset are described in Safikhani *et al*.^9^. Molecular information for the gCSI dataset was taken from the supplementary data of the publication^4^.

#### Cell line drug response data

Drug response data included three metrics: IC_50_, AUC, and viability at 1 µM (Fig. s1). IC_50_ (half maximal inhibitory concentration) and AUC (area under the drug response curve) information was obtained from the PharmacoGx package; in particular we used values recomputed by the package from the raw data ‒ “ic50_recomputed” and “auc_recomputed”. In order to handle outlier values in the IC_50_ data, we truncated the distribution of IC_50_ values at the 85th percentile for each drug. Viability at 1 µM values were calculated from the raw drug response data in CCLE, CTRP and GDSC datasets.

#### Cell line tissue type and division rate data

For tissue type and division rate predictions in gCSI dataset we used tissue labels and division rate values from the cell line meta-information provided in the supplementary data of the publication^4^.

#### Xenograft molecular data

Molecular data included gene expression, copy number information, and mutation information. Expression data comes as continuous FPKM (fragments per kilobase of transcript per million mapped reads) values from RNA-seq. Copy number information comes from SNP arrays, it’s continuous. Mutation information has been derived from RNA-seq data and represented by binary values per gene (1/0 for presence/absence of mutation). All molecular data was taken from the supplementary data of the publication^25^.

#### Xenograft drug response data

We tested 4 different drug response metrics derived from raw drug response data i.e. volume change of the tumour during the treatment course between day 0 and day 21: tumour volume (at day 21), integral response (difference between the areas under the tumor growth curves from control and treated mice: AUC_control_ − AUC_treated_), slope of the tumor growth curve, and differential slope (difference between the slopes under the tumor growth curves from control and treated mice: slope_control_ − slope_treated_), see Fig. s2. Raw drug response data was taken from papers’ supplementary data^25^.

#### Xenograft tissue type and slope of growth curve data

For tissue type prediction in xenografts we used tissue labels from paper’s supplementary data^25^. Slope of the tumor growth curve rate for untreated mice was calculated from raw drug response data (control cases).

#### Sample sizes

The sample size for each modelling task is provided in the Supplementary Tables s2 and s3.

### Modelling

#### Classes of supervised learning

Drug response prediction tasks and division rate/slope of growth curve prediction were regression tasks. Tissue type prediction tasks were classification tasks.

#### Modelling methods and hyperparameters

For regression tasks we used two modelling methods: support vector machine (svmRadial) and random forest (rf) in the first and second part of our analysis, respectively. For classification tasks we used xgBoost (xgbTree)^27^.

Each modelling method has its own set of hyperparameters: svmRadial ‒ sigma and C (cost); random forest ‒ mtry; xgbTree ‒ nrounds, max_depth, eta, gamma, colsample_bytree, min_child_weight, subsample.

#### Feature selection

In order to select a subset of all available features for modelling we performed feature selection using only data from the training set. We evaluated each feature individually with respect to the association between a feature vector and a vector with target variables (filter feature selection). For this evaluation we used two functions from the caret package ‒ gamScores for regression tasks and anovaScores for classification tasks.

The function gamScores fits a generalized additive model between a single predictor and the outcome using a smoothing spline basis function. For classification, anovaScores treats the outcome as the independent variable and the predictor as the outcome. In this way, the null hypothesis is that the mean predictor values are equal across the different classes. In each function a p-value for the whole model F-test is returned and is used as the score^28^.

#### Accuracy metrics

For regression tasks we used three accuracy metrics: root of mean squared error (RMSE), coefficient of determination (R^2^), and concordance index. The concordance index is the rank correlation between observed and predicted data^29^. RMSE and R^2^ were calculated using the postResample function from caret package, the concordance index was calculated using “concordance.index” function from survcomp package (version 1.28.5)^30^.

For classification tasks we used the percentage of correctly predicted samples as the accuracy metric.

#### Model training and evaluation procedure

The following procedure was used for model testing for each [drug, dataset, drug response metric] combination (see Fig. s3):

1. We split the data into training (70%) and test (30%) sets.
2. Using the training set we performed feature selection.
3. Then we fitted the model with N (from 10 to 500) selected features (with lowest p-values) on the training set data. In order to select hyperparameters, 30 different combinations of them were tested on training data via cross-validation procedures, and then the combination of hyperparameters that provides the best accuracy was used for fitting the final model.
4. We applied the model to the test set, and calculated the accuracy metrics.
5. We repeated steps (1–4) ten times and got average values of accuracy metrics.

## Results

### Testing influence of different aspects of model training on prediction accuracy

In order to find an optimal strategy for training drug response models, we explored the model parameter space in terms of (1) molecular types of features: expression only vs. expression + copy number + mutation; (2) metrics of drug response: IC_50_, AUC, Viability_1 µM; and (3) number of features used in the model: 10, 50, 200, and 500. To test these options we built a number of models for seven drugs ‒ erlotinib (EGFR), paclitaxel (*β*-tubulin), lapatinib (EGFR), PLX4720 (RAF), sorafenib (RAF), nutlin-3 (MDM2) and nilotinib (ABL/BCR-ABL). These seven drugs were selected because they were in overlap between CCLE, CTRP, and GDSC screens.

**Figure 2:**
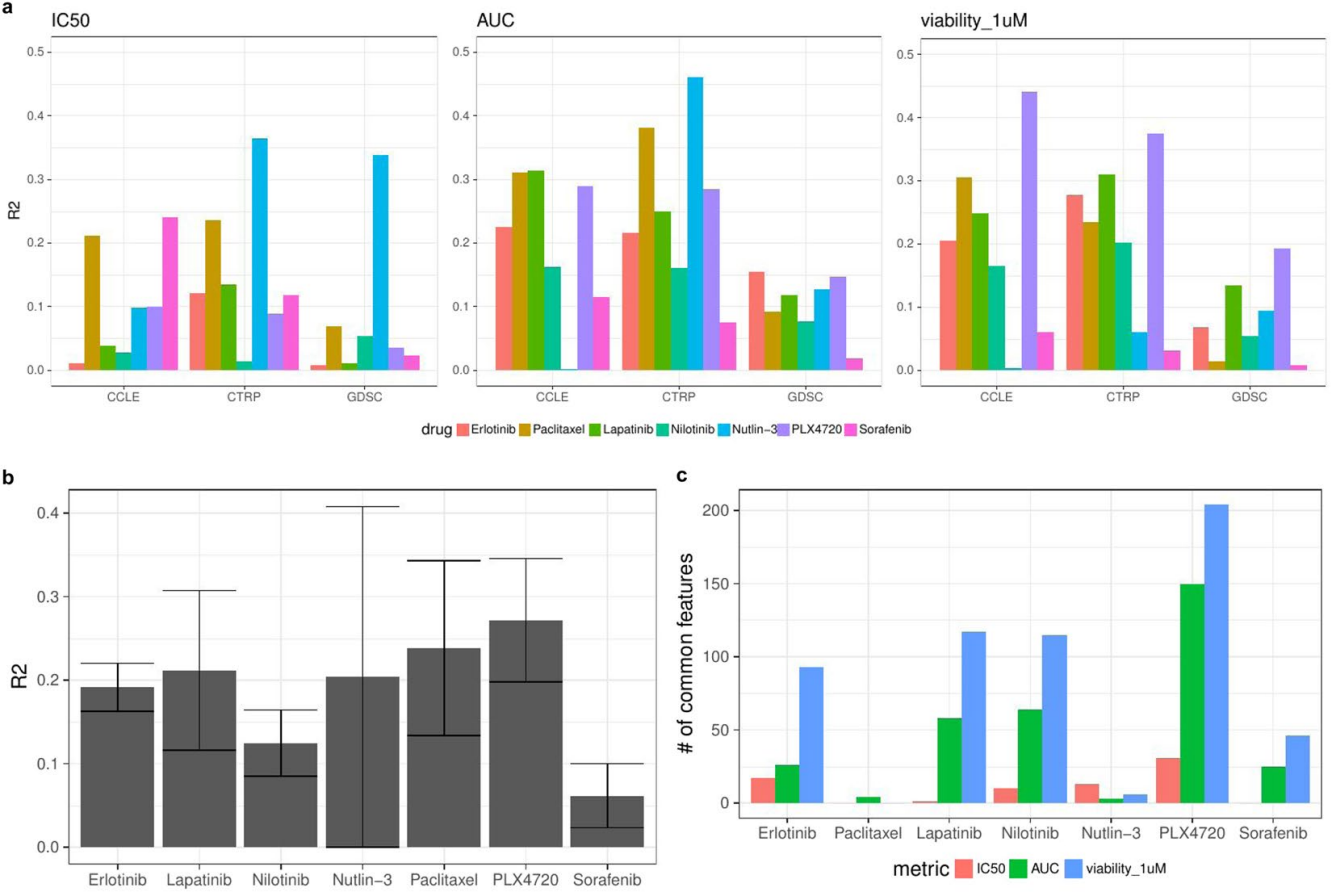
(**a**) R^2^ for 7 drugs across all tests, number of variables = 500. (**b**) Average R^2^ values (across three datasets) for each drug separately (for models with AUC metric). Error bars depict ± one standard deviation. (**c**) Number of common features out of the top 500 features between 3 datasets (CCLE, CTRP and GDSC) for each drug within each drug response metric.

Results (for tests with all genomic features) in terms of R^2^ and concordance index are shown in Figs. 2a and s4, respectively. Each plot shows results for a certain response metric combination, and within each plot there are results for each drug in each data set for the tests with 500 variables (for the results with other variable set sizes see Fig. s5).

In order to provide a reference for R^2^ values we repeated the analysis for Lapatininb using a linear regression model with only one predictor ‒ expression of the ERBB2 gene, which is a known biomarker for Lapatinib response in breast cancer^31^. The resulting average R^2^ values were 0.04, 0.25 and 0.27 for IC_50_, AUC, and viability_1 µM metrics, respectively.

**Figure 3:**
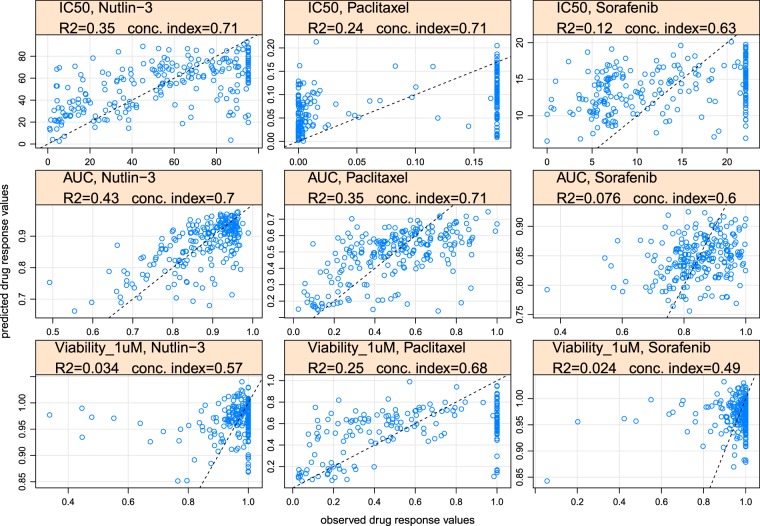
Observed vs. predicted values for Nutlin-3, Paclitaxel and Sorafenib. Rows from top to bottom correspond to the IC_50_, AUC, viability at 1µM metric, respectively. Data from the CTRP dataset is used for model training and testing.

We plotted predictions against observed values for each drug response metric for Nutlin-3, Paclitaxel and Sorafenib. In each plot the data points correspond to a test set from one particular (random) train/test split (Figs. 3 and s6). A dotted line shows where data points should lie in the ideal case of 100% correct predictions. In cases where the accuracy of prediction is satisfactory, the data points are grouped around the dotted line, e.g. IC_50_, nutlin-3 (1^st^ row, 1^st^ column); AUC, nutlin-3 and paclitaxel (2^nd^ row, 1^st^ and 2^nd^ columns); viability_1uM, paclitaxel(3^rd^ row, 2^nd^ column). In cases where prediction accuracy is low, predicted and observed values are hardly correlated and therefore data points are not grouped around the dotted line.

In these tests the average value of R^2^ for modelling with all genomics data (0.153) was just slightly higher than for modelling with only expression data (0.145). While these differences are small for most of the drugs, for nutlin-3 and PLX4720 they are a bit more pronounced (Fig. s7). Below, we will discuss only models that are based on all genomic features (expression, copy number and mutation values).

We have not observed correlation between the number of features selected for modelling and the accuracy of prediction within the tested range of features (Figs. s5 and s12).

We found differences in accuracy for models based on different drug response metrics, IC_50_, AUC, and Viability_1uM: R^2^_IC50_ = 0.111, R^2^_AUC_ = 0.186, R^2^_viability_1uM_ = 0.162 (These differences are significant for comparisons IC50 vs. AUC and IC50 vs. Viability_1uM, p-values from t-test are 2.4∙10^−5^ and 4.3∙10^−3^, respectively. Difference for the comparison AUC vs. Viability_1uM is not significant, p-value is 0.2).

We checked the number of common features out of the selected top 500 features for each drug between 3 datasets (CCLE, CTRP and GDSC) within each drug response metric. The number of common features is relatively small for the IC_50_ metric (average = 10) and higher for AUC and viability at 1µM metrics (average = 47 and 83 respectively, see Fig. 2c). Independently of the response metric used, there is almost no common features across paclitaxel models and across nutlin-3 models.

The average R^2^ and concordance index values for each drug (for AUC models) are shown in Figs. 2b and s8. Five drugs ‒ PLX4720, paclitaxel, lapatinib, nutlin-3 and erlotinib had average R^2^ between 0.2 and 0.3, while nilotinib and sorafenib showed the lowest average predictability (R^2^ = 0.12 and 0.06, respectively). Average R^2^ for each tissue separately is shown in Fig. s9.

### Comparison in accuracy between our method and methods from CCLE and DREAM Challenge

We compared our results for the CCLE dataset with the performance of elastic net models from the original CCLE study^1^ and with the performance from integrated (combined) random forest method (CRF)^32^, which was the second top performing method in the DREAM drug response prediction challenge^33^ (We are comparing our results with the second top performing method instead of the first one simply because both methods have quite similar accuracy score in the original paper, wpc-index equals to 0.583 and 0.577 correspondingly, but the second method to our convenience was already tested on the CCLE dataset with essentially the same accuracy metric that we are using in our analysis). Corresponding R^2^ values are shown in Table 2.

**Table 2:** Comparison between prediction results from different methods in the form of R^2^ values.

Drug	Elastic Net	CRF-400	CRF-20000	SVM-500 (our results)
erlotinib	0.09	0.16	0.18	**0**.**22**
paclitaxel	**0**.**36**	0.30	0.30	0.31
lapatinib	0.20	0.30	0.28	**0**.**31**
nilotinib	**0**.**58**	0.30	0.30	0.16
nutlin-3	0.01	0.08	**0**.**10**	0.003
plx4720	**0**.**30**	0.20	0.23	0.29
sorafenib	0.07	0.17	**0**.**22**	0.12

Dataset: CCLE. Response metric: AUC. Elastic net denotes the approach used in^1^. CRF-200 and CRF-2000 denote the approach used in^32^. SVM-500 denotes our results. The highest R^2^ value for each drug is highlighted in boldface.

### Tissue type, doubling time and drug response prediction in cell lines and xenografts

We used the gCSI study^4^ as an *in-vitro* training set. It is a high-quality pharmacogenomic dataset reasonably consistent with the CCLE and GDSC datasets. We used the NIBR PDXE^25^ data as an *in-vivo* validation set since this is the only publicly available xenograft screen. We assessed 6 modelling tasks in each set, i.e. prediction of tissue type, doubling time/slope of the tumor growth curve, erlotinib response (lung samples), gemcitabine response (pancreas samples), paclitaxel response (breast samples), and paclitaxel response (lung samples). Tissue type and doubling time prediction tasks serve as a positive controls ‒ we assume that these phenotypes should be explained by genomics data better than drug response.

For tissue prediction and doubling time/slope of the untreated tumor growth curve we used the top 400 features with the lowest p-values from F-test (see Feature selection subsection in Data and Methods section). For drug response prediction (since sample sizes were much lower) we used just the top 100 features.

In the xenograft tissue prediction we had 5 tissue classes with 27–50 samples per tissue. In the cell line tissue prediction we tried modelling with 13 tissue-classes with 10–68 samples per tissue, and with the 6 largest tissue classes (each tissue had at least 23 samples).

Prediction accuracy results for all prediction tasks are collected in Table 3. For tissue type prediction we report the percentage of correctly predicted samples, for doubling time/slope and drug response prediction we report R^2^ and concordance index values.

**Table 3:** Prediction accuracy for all prediction tasks.

	Tissue type (Accuracy)	Doubling time (cell lines)/slope of the growth curve (xenografts) (R^2^, concordance index)	Drug response (R^2^, concordance index)					
			*drug response metric*	erlotinib (EGFR) Lung 68 lines 25 xen.	gemcitabine (DNA synth.) Pancreas 26 lines 32 xen.	paclitaxel (*β*- tubulin) Breast 29 lines 38 xen.	paclitaxel (*β*- tubulin) Lung 68 lines 23 xen.	Average across 4 drugs
Cell lines (gCSI) 329 samples	Acc_6tissues_ = **0**.**79** Acc_13tissues_ = **0**.**64**	**0**.**17** (0.64)	AUC	**0**.**06** (0.57)	**0**.**13** (0.61)	**0**.**14** (0.66)	**0**.**08** (0.57)	**0**.**10** (0.60)
Xenografts (NIBR) 191 samples, 23–38 samples per drug	Acc = **0**.**89**	**0**.**19** (0.60)	Tumor volume	**0**.**34** (0.69)	**0**.**04** (0.49)	**0**.**08** (0.53)	**0**.**46** (0.74)	**0**.**23** (0.61)
			Integral response	**0**.**18** (0.59)	**0**.**03** (0.50)	**0**.**08** (0.57)	**0**.**09** (0.47)	**0**.**10** (0.53)
			slope	**0**.**31** (0.65)	**0**.**15** (0.54)	**0**.**11** (0.54)	**0**.**44** (0.63)	**0**.**25** (0.59)
			Differential slope	**0**.**12** (0.50)	**0**.**09** (0.53)	**0**.**10** (0.54)	**0**.**27** (0.35)	**0**.**15** (0.46)

We report here accuracy (percentage of correctly predicted samples) for tissue prediction, and R^2^ with concordance index for doubling time/slope and drug response prediction (R^2^ values are in bold font, concordance index values are in round brackets). Each column corresponds to a certain prediction task ‒ tissue type, doubling time/slope of the growth curve or drug response (4 tasks for four different drug-cohort groups). Two main rows correspond to cell lines and xenograft results, while the second row (xenograft results) is subdivided into 4 additional rows, each for different xenograft drug response metrics.

**Figure 4:**
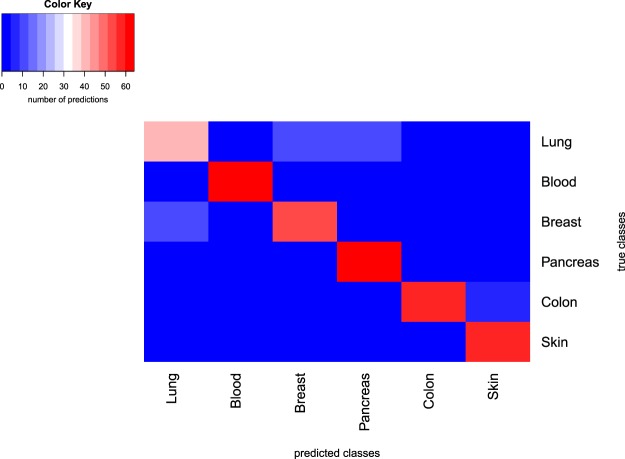
Confusion table heatmap for results of tissue classification in cell lines. Row labels depict true classes, column labels depict predicted classes. The test set contained 7 samples for each tissue, and the modelling procedure was repeated 10 times, so for each tissue class 70 predictions were made. The color shows the number of predicted classes per each true class.

In addition to tissue prediction tests performed using all available cell lines and xenografts we made a test with equal number of samples per tissue (16 samples in training set and 7 samples in test set). A heatmap of the confusion table for the case of cell line predictions is shown in Fig. 4.

While the differences in accuracies between tissue type prediction and doubling time/slope prediction are consistent for cell lines and xenografts, drug response prediction accuracies for the same drugs are not consistent between cell lines and xenografts. Particularly, the best performing drugs are gemcitabine and paclitaxel (breast) for cell lines and erlotinib, paclitaxel (lung) for xenografts. The level of consistency is also illustrated by the number of common molecular features between cell lines and xenografts out of the top features pre-selected for modelling. There are 31 common features out of the top 400 for tissue prediction between cell lines and xenografts, only 4 common features out of the top 400 for doubling time/slope prediction, and almost no common features out of the top 100 for drug response prediction.

**Figure 5:**
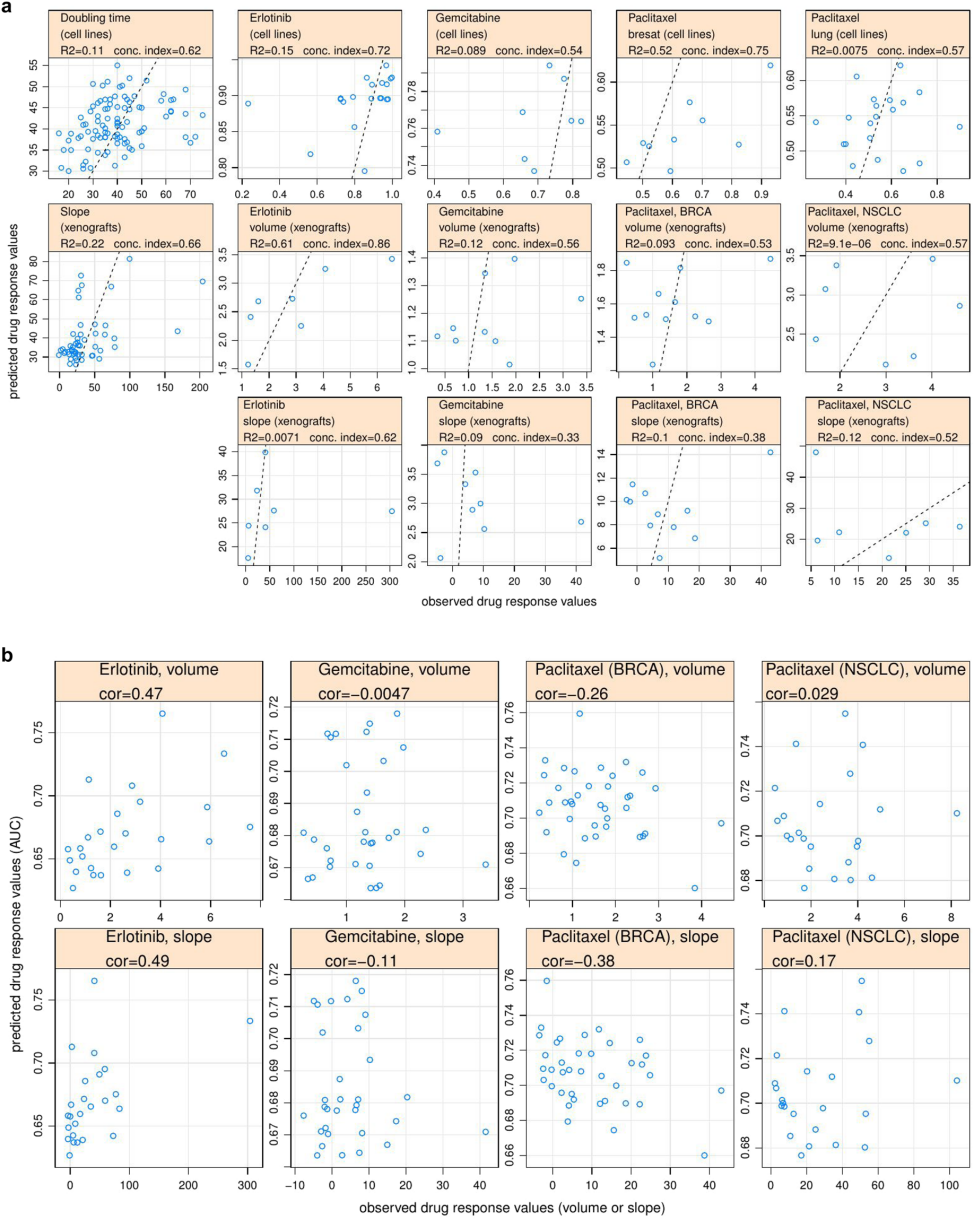
(**a**) Observed vs. predicted values for different regression tasks. (**b**) Observed vs. predicted values for [cell line → xenograft] prediction and corresponding correlation coefficients. Only results for tumour volume and slope response metrics are shown.

In order to make the quality of prediction across different regression prediction tasks visually accessible, we plotted observed versus predicted values for different prediction tasks. In each task the data points correspond to a test set from one particular (random) train/test sets split (Fig. 5a).

Manual inspection of outlier cases from observed vs. predicted plots (Fig. 3a) shows that often a model’s inability to provide an accurate prediction for certain samples (outliers) is driven by under-representation of samples with similar molecular characteristics to these outliers in the training set.

### Cross-prediction between cell lines and xenografts

We tested how well the models trained on cell line data can explain drug response in xenografts. For that we 1) trained drug response models using cell line genomics and drug response data (AUC), 2) got model predictions using xenograft molecular data as inputs, 3) assessed the correlation of resulting predictions (in cell line AUC units) with the actual xenograft drug response. We tried two strategies with respect to training set composition ‒ training using all cell lines and training using only cell lines that match the tissue type of the corresponding set of xenograft samples. Results in terms of correlation coefficients as well as predicted vs. observed plots for this analysis (for the case where we used all cell lines for training) are shown in Figs. 5b and s10.

Among four xenograft response metrics used in this study, volume and slope are expected to be positively correlated with cell line AUC while integral response (ΔAUC) and differential slope are expected to be negatively correlated. For both training strategies (all cell lines or cell lines from one relevant tissue type) we managed only for erlotinib to get predictions with the right sign of correlation coefficient (between predictions and observed drug response) and substantial absolute value for all xenograft drug response metrics. Volume and slope metrics worked especially well in the case of erlotinib, with correlation about 0.5 in both cases.

## Discussion

We have shown that, dependent on the cellular phenotype that we want to predict from genomic data, the accuracy of prediction varies substantially. Tissue type classification can be achieved with relatively high accuracy. The percentage of correctly predicted samples is 0.79 for the cell line set, and 0.89 for the xenograft set. The accuracy of prediction depends substantially on the size of the training set for each tissue class. When we additionally include tissues which have from 10 to 20 samples into our cell line modelling set, the average accuracy drops from 0.79 to 0.64.

While the accuracy of tissue type classification is high on average, it varies across tissues. In a series of tests where we use equal number of samples per tissue (16 samples in the training set and 7 samples in the test set), we found that we have the lowest accuracy for lung samples, higher accuracy for breast samples, and the highest accuracy for pancreas, colon, skin and blood samples (Fig. 4). Interestingly this ranking holds for both cell line and xenograft predictions. A fraction of lung samples is often misclassified as breast samples (and to a lesser extent the other way around) which results in comparatively lower accuracies for lung samples. This can be explained by the partial overlap between features that separate lung and breast samples from other samples, particularly expression of some transcription regulators genes and membrane protein genes (see Table s1 for details). On a PCA plot the cluster of lung samples has a big overlap with the cluster of breast samples (see Fig. s11). The ability to predict tissue type using cellular expression data assures that more complex phenotypes can be in principle predicted from expression data as well.

The second cellular phenotype we aimed to predict was the cell line doubling time, or slope of untreated tumor growth curve in case of xenografts. Here we got lower accuracy compared to the tissue type prediction. The average accuracy is quite consistent between cell lines and xenografts: R^2^_cell lines_ = 0.17, R^2^_xenografts_ = 0.19. The lower accuracy of prediction shows that, unlike for tissue type, there is less information about the rate of cell division in the static expression data, which can be due to the post-translational regulation of the activity of cell cycle proteins. Additionally the prediction problem is complicated by the fact that expression data originates from the pooled group of cells that were at different cell cycle stages prior to expression profiling.

The main phenotype of interest in our analysis is drug response. Analogously to the doubling time prediction case, the accuracy of drug response prediction is substantially lower than accuracy of tissue type prediction. A possible reason for that is (as in the case of doubling time) that the static gene expression data obtained prior to drug treatment doesn’t necessarily contain enough information to fully explain drug response (although high expression of a drug’s target gene is a good biomarker of response, in the case when high expression of this gene is essential for cell proliferation).

We found that accuracy of drug response prediction doesn’t strongly depend on a choice of machine learning algorithm. If we look at models for erlotinib response in cell lines, the average R^2^ for random forest models (tested on gCSI data) is comparable to average R^2^ for support vector machine models (tested on data from CCLE, CTRP and GDSC): R^2^_RF_ = 0.23, R^2^_SVM_ = 0.19. Also according to our results the number of top features selected for modelling (via filter feature selection), within the tested range of 10–5000 features, doesn’t influence the resulting accuracy of predictions, which rather depends on the strength of correlation between top feature(s) and the outcome (Figs. s5 and s12).

We see that the accuracy of drug response prediction varies across the drugs, e.g. in our tests based on CCLE, CTRP and GDSC datasets average R^2^ ranges from 0.06 for Sorafenib to 0.27 for PLX4720 (results for AUC metric).

We found that the average accuracy of models based on all genomic data (expression + mutation + copy number data) is just marginally higher than the average accuracy of models based on expression data only (Fig. s7), which shows that expression data can explain most of the variation in drug response. This is consistent with the finding from the DREAM drug response prediction challenge^33^. However, the use of additional omics profiles, e.g. methylation and proteomics data can still improve the accuracy of prediction^34^. Also it was recently shown that transcriptional perturbation signatures (e.g. from the LINCS-L1000 project^35^) can be successfully used for drug response prediction^36^.

The choice of a drug response characterization metric has a serious impact on the accuracy of predictions. Having compared three drug response metrics for cell lines we found that the area under the drug response curve (AUC) provides the highest predictive performance. AUC combines information about drug efficacy and potency into a single value, and it was reported to be the most robust metric previously^37^. Additionally we compared these three metrics with a recently proposed GR_AOC metric which takes into account the difference in growth rates between cell lines^38^. GR_AOC performed slightly worse than AUC and viability_1uM but better than IC_50_ (Fig. s13). Comparing performance of different xenograft drug response metrics we found that simple metrics like “tumor volume (day 21)” and “slope” perform better (average R^2^ = 0.23 and 0.25) than those which additionally take into account data from untreated controls ‒ “Integral response” and “Differential slope”, with an average R^2^ = 0.095 and 0.145, respectively.

We also showed that the size of the training set is an important determinant for the accuracy of a model. In our tests based on gCSI data the average R^2^ for models trained on cell lines from all tissues (n = 329) was 0.267, while for models that used only cell lines from a certain tissue for each drug (n = 26–68), the average R^2^ was 0.102. Low number of samples makes it harder to distinguish the true signal from noise^33^, especially in the case of multi-omics datasets which are high-dimensional. To deal with high-dimensionality in our modelling tests, we employed filter-based feature selection (i.e. selected a subset of features that have high association with a target variable). Alternative options for combating high-dimensionality include literature based feature selection (e.g. using the list of cancer census genes from COSMIC for modelling^24^), and common dimensionality reduction methods like PCA. Creating aggregated features using pathway information^39^ can help with dimensionality reduction, however in our preliminary tests we found that combining pathway features with simple genomic features (selected via feature selection) can improve resulting accuracy only marginally (see Fig. s14).

Finally we assessed our ability to predict drug response in xenografts using models trained on cell line data. We tested our predictions in four cohorts ‒ NSCLC treated with erlotinib, PDAC treated with gemcitabine, BRCA and NSCLC treated with paclitaxel. Only for the NSCLC cohort treated with Erlotinib our predictions were moderately accurate, i.e. positively correlated (r = 0.5) with the tumor volume and the slope of the tumor growth curve. Performance of the models built and tested on xenograft and cell line data separately can’t explain why this type of prediction worked only for erlotinib-treated cohort. Indirectly it shows that pharmacogenomic associations were consistent between cell line and xenograft dataset only in the case of erlotinib, which has only one target-molecule, EGFR, but not in the cases of more pleiotropically acting drugs such as gemcitabine and paclitaxel which block DNA synthesis and cell division, respectively. Also differences in cell growth and differences in drug response quantification between 2D and 3D model systems probably contributed to the inconsistency in pharmacogenomics associations between cell lines and xenografts.

## Conclusions

While more and more data from high-throughput drug sensitivity screenings become available, accurate drug response prediction remains challenging. As we show in the present study, we cannot expect that accuracy of drug response prediction in cell lines (or xenografts) will be equal (or even close) to accuracy of tissue type prediction. Nevertheless it is possible to fit models of drug response which will provide moderate accuracy. Here we explored the parameter complexity one faces when fitting these models. We found that generally the choice of a particular machine learning algorithm doesn’t influence accuracy, and the number of variables included in the model doesn’t matter much. What matters, though, is the degree of association between the top predictor variables and outcome. Among the parameters that do influence accuracy are class of molecular predictors ‒ we see that expression data has the highest explanatory power, response metric (AUC provide the best results in cell lines, Volume and Slope of tumor growth curve demonstrate the best results in xenografts) and, quite importantly, the number of samples in the training set.

Mastering cell line drug response models will make it ultimately possible to perform personalized prediction of drug sensitivities for individual cancer patients^40^. In the present study we tried to predict drug response in xenografts using models trained on cell line data, which can be seen as an approximation to the patient prediction scenario. We managed to get reasonably accurate predictions only in the NSCLC xenograft cohort treated with erlotinib, but not in other cohorts treated with either gemcitabine or paclitaxel. With more xenografts and patient material screens publically available in the future, it will be possible to understand in which cases drug response associations are transferable between cell lines and xenografts/patients and in which cases they are not. This understanding, combined with the wealth of available high-throughput data, will bring closer the era of personalised cancer medicine.

## Supplementary information

Supplementary materials.

## Data Availability

Our work complies with the guidelines for research reproducibility from Gentleman *et al*.^41^. The analysis code and its documentation is open-source and freely available from URLs https://github.com/RomaHD/DrugRespPrediction and 10.5281/zenodo.3626896. The *in vitro* pharmacogenomic data can be obtained from the PharmacoGx package^26^ directly. *In vivo* pharmacogenomic data are available from the Journal’s website^25^.
