# Supplementary material for: Assessment of modelling strategies for drug response prediction in cell lines and xenografts
Source: Sci Rep. 2020 Feb 18;10:2849. doi: 10.1038/s41598-020-59656-2 (PMC7028927; doi:10.1038/s41598-020-59656-2)
Supplement: Supplementary file 1 — Supplementary materials. [file 41598_2020_59656_MOESM1_ESM.pdf]

Supplementary materials

Assessment of modelling strategies for drug response prediction in cell lines and xenografts

Roman Kurilov, Benjamin Haibe-Kains, Benedikt Brors

Cell line drug response metrics.

Cell line drug response data included three metrics: IC_{50}, AUC and viability at 1 μM (Fig. s0).
IC_{50} (half maximal inhibitory concentration) metric values were obtained via PharmacoGx package. Particularly "ic50_recomputed" values were used. In order to estimate IC_{50} dose response curves were fitted on raw viability data to the equation:

$$y = E_\infty + \frac{1 - E_\infty}{1 + (\frac{x}{IC50})^{HS}}$$

"where the maximum viability is normalized to be y = y(0) = 1, E∞ denotes the minimum possible viability achieved by administering any amount of the drug, IC_{50} is the concentration at which viability is reduced to half of the viability observed in the presence of an arbitrarily large concentration of drug, and HS is a parameter describing the cooperativity of binding. HS < 1 denotes negative binding cooperativity, HS = 1 denotes noncooperative binding, and HS > 1 denotes positive binding cooperativity. The parameters of the curves are fitted using the least squares optimization framework" (Smirnov, P., et al. "PharmacoGx: an R package for analysis of large pharmacogenomic datasets." *Bioinformatics* 32.8 (2015): 1244-1246.)
In order to handle outlier values in IC_{50} data we truncated the distribution of IC_{50} values at the 85th percentile for each drug.
AUC (area under the drug response curve) metric values were also obtained via PharmacoGx package. Particularly "auc_recomputed" values were used. AUC values are calculated as area *above* the drug response curve fitted to the data (see IC_{50} section). In order to get the actual area *under* the curve we then subtracted obtained values from 1 (the total area):
AUC_final = 1 - AUC_recomputed
Viability at 1 μM metric values were calculated by fitting logistic regression drug response curve on the raw viability data using nplr package (Commo, F., and Briant M.B.. "R package nplr n-parameter logistic regressions." *V. 0.1–7* (2016).) and taking the curve's value at 1uM.

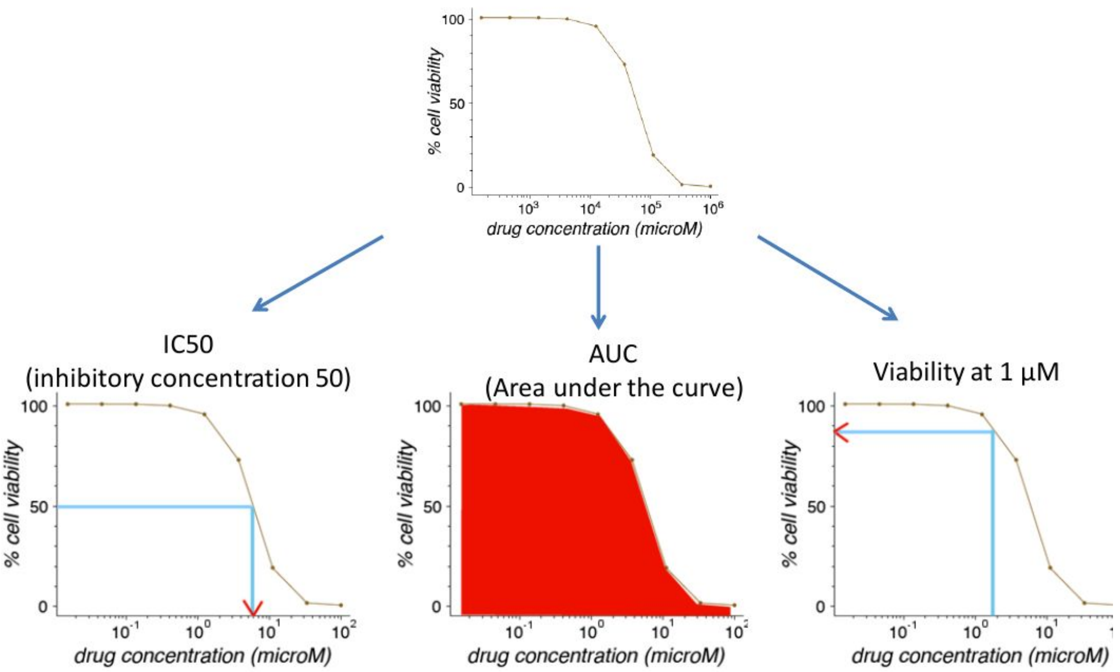

Figure s1. Cell line drug response metrics. Figure depicts raw drug response data and three derived metrics, IC50, Area under the curve, and Viability at 1uM.

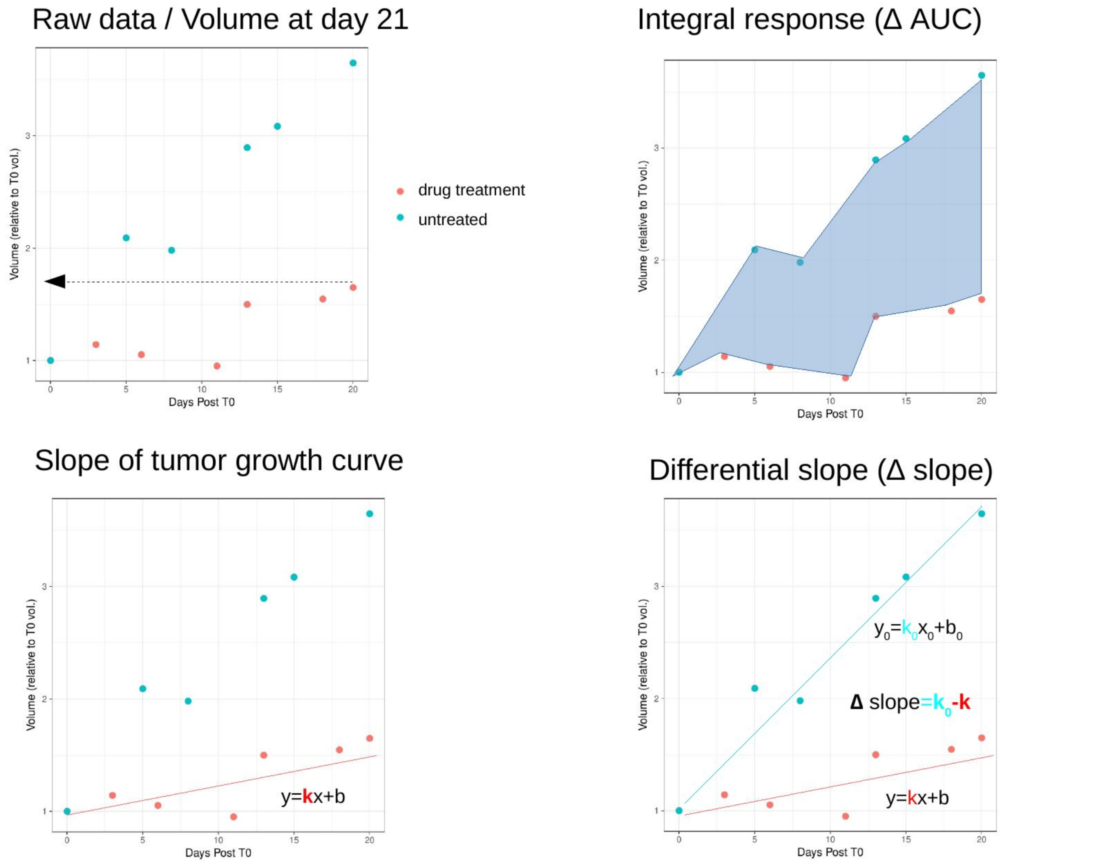

Figure s2. Xenograft's drug response metrics. Figure depicts four drug response metrics: Volume (day 21), Slope of tumor growth curve, Integral response, and Differential slope.

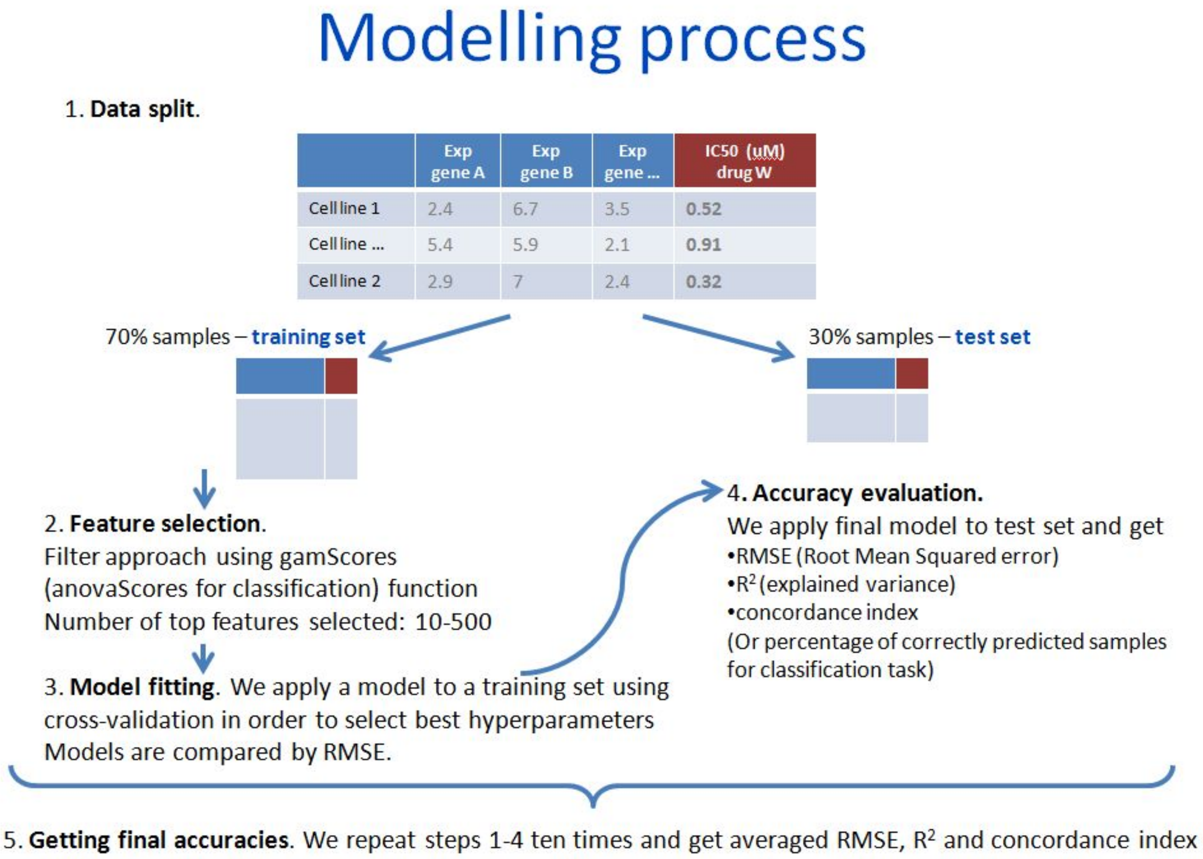

Figure s3. Steps of the modelling process. 1. Data split. 2. Feature selection. 3. Model fitting. 4. Accuracy evaluation.

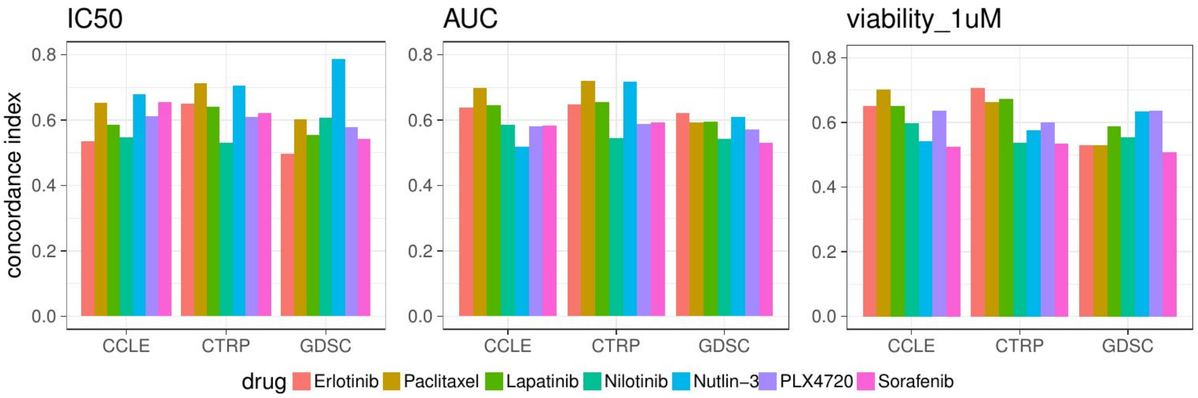

Figure s4. (**a**) Concordance index for 7 drugs across multiple testing conditions, number of variables=500.

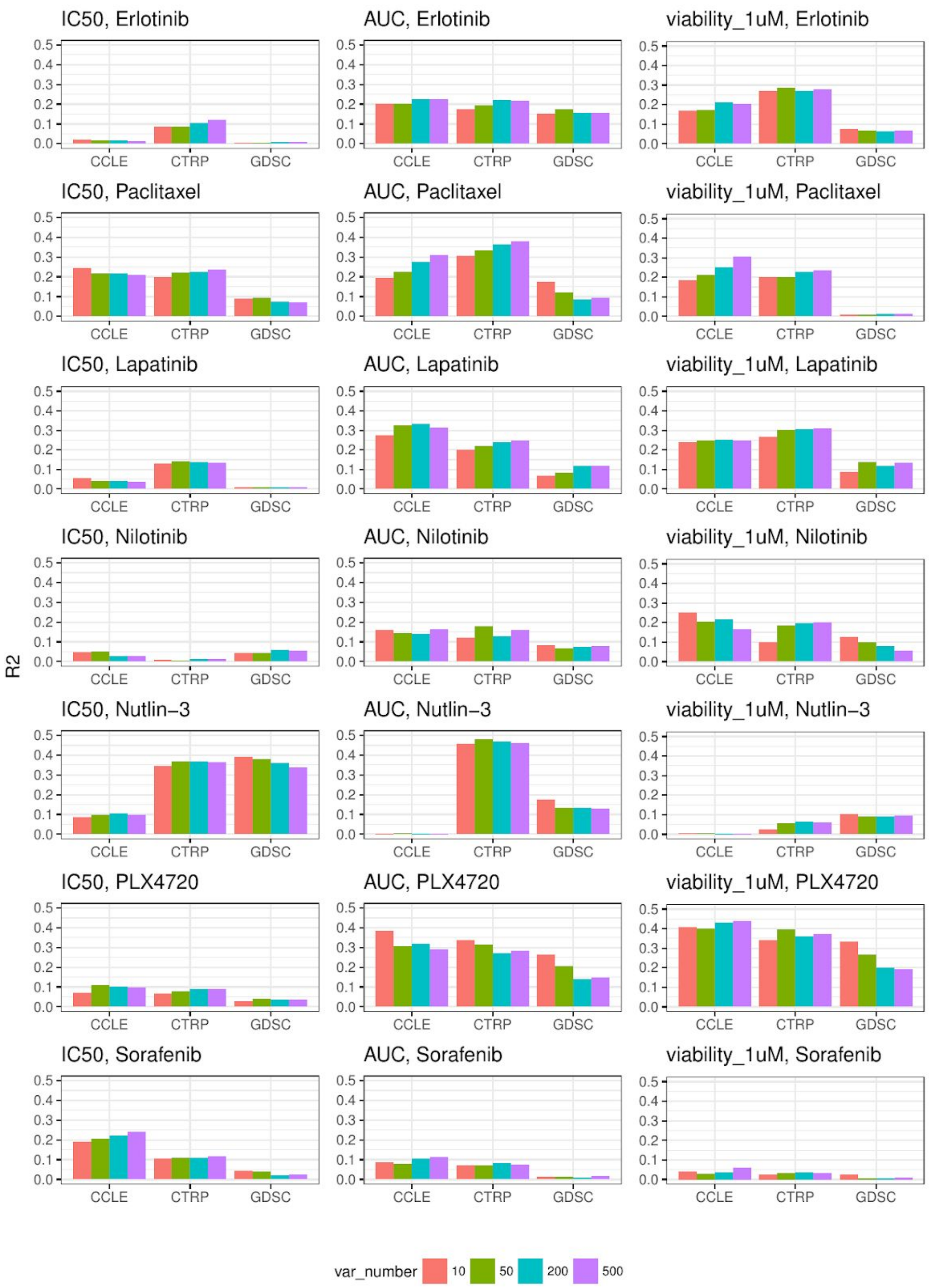

Figure s5. R^2 for 7 drugs across multiple testing conditions. Rows represent different drug response metrics, IC50, AUC, Viability at 1μM, columns represent different drugs. On each plot there results for three tested datasets: CCLE, CTRP, GDSC. Color coding reflects number of variables in the model.

a

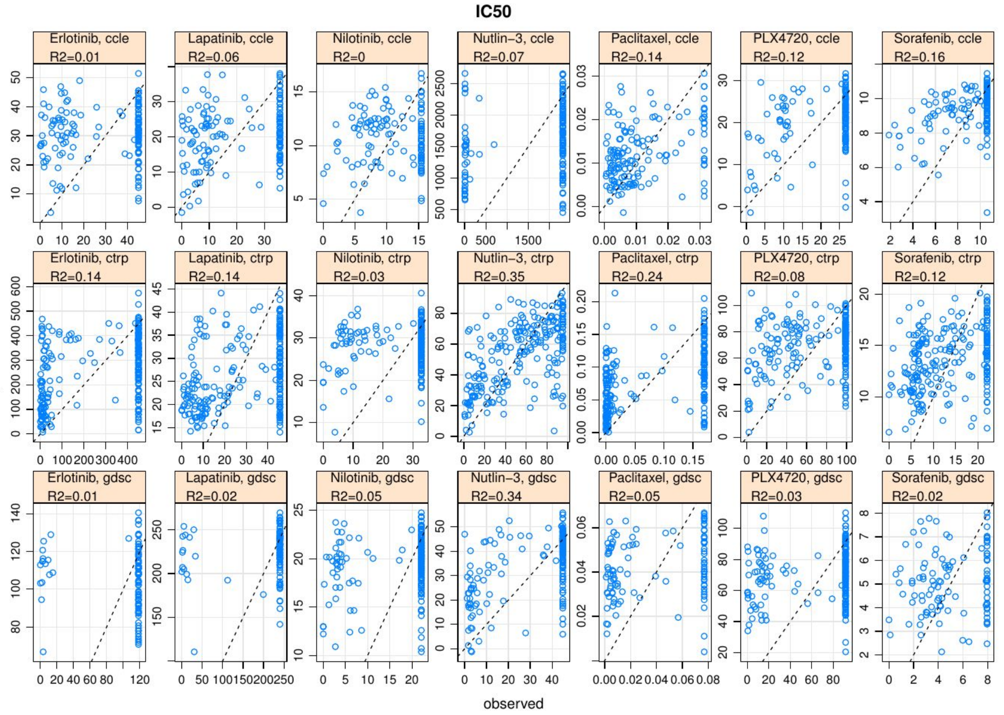
IC50
Erlotinib, ccle R2=0.01
Lapatinib, ccle R2=0.06
Nilotinib, ccle R2=0
Nutlin-3, ccle R2=0.07
Paclitaxel, ccle R2=0.14
PLX4720, ccle R2=0.12
Sorafenib, ccle R2=0.16
Erlotinib, ctrp R2=0.14
Lapatinib, ctrp R2=0.14
Nilotinib, ctrp R2=0.03
Nutlin-3, ctrp R2=0.35
Paclitaxel, ctrp R2=0.24
PLX4720, ctrp R2=0.08
Sorafenib, ctrp R2=0.12
Erlotinib, gdsc R2=0.01
Lapatinib, gdsc R2=0.02
Nilotinib, gdsc R2=0.05
Nutlin-3, gdsc R2=0.34
Paclitaxel, gdsc R2=0.05
PLX4720, gdsc R2=0.03
Sorafenib, gdsc R2=0.02
predicted
observed

b

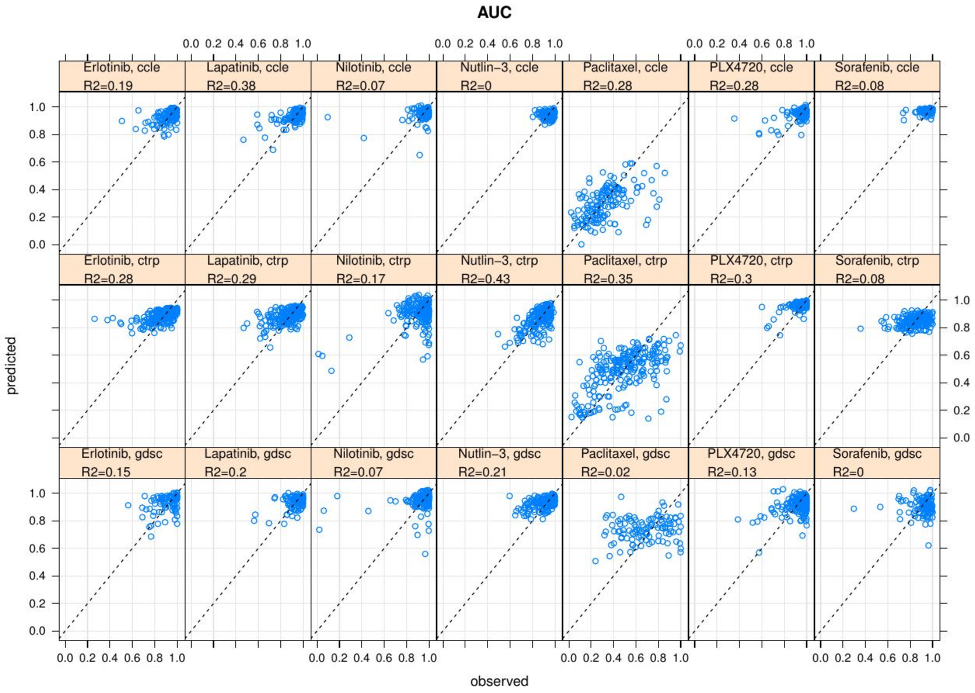
AUC
Erlotinib, ccle R2=0.19
Lapatinib, ccle R2=0.38
Nilotinib, ccle R2=0.07
Nutlin-3, ccle R2=0
Paclitaxel, ccle R2=0.28
PLX4720, ccle R2=0.28
Sorafenib, ccle R2=0.08
Erlotinib, ctrp R2=0.28
Lapatinib, ctrp R2=0.29
Nilotinib, ctrp R2=0.17
Nutlin-3, ctrp R2=0.43
Paclitaxel, ctrp R2=0.35
PLX4720, ctrp R2=0.3
Sorafenib, ctrp R2=0.08
Erlotinib, gdsc R2=0.15
Lapatinib, gdsc R2=0.2
Nilotinib, gdsc R2=0.07
Nutlin-3, gdsc R2=0.21
Paclitaxel, gdsc R2=0.02
PLX4720, gdsc R2=0.13
Sorafenib, gdsc R2=0
predicted
observed

c

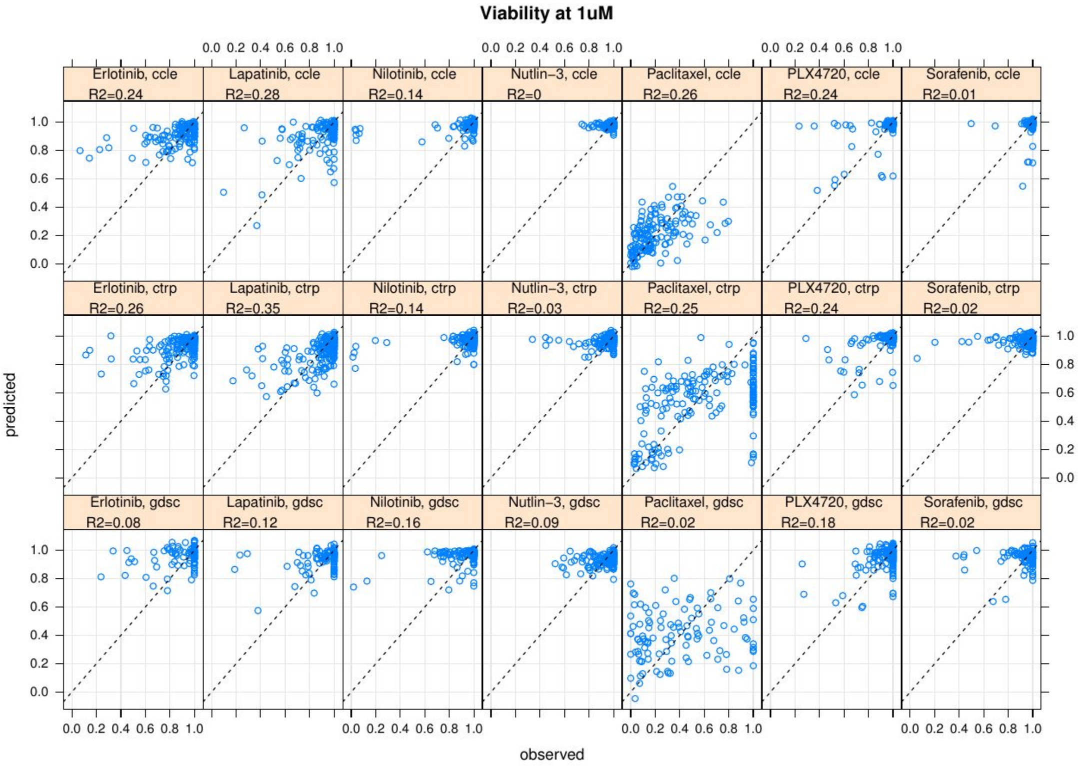

Figure s6. Observed vs. predicted values for different drugs/datasets combinations within each drug response metric (IC50, AUC, viability at 1μM). On each plot columns represent different datasets, rows represent different drugs. **(a)** IC50 values. **(b)** AUC values. **(c)** viability_1uM values.

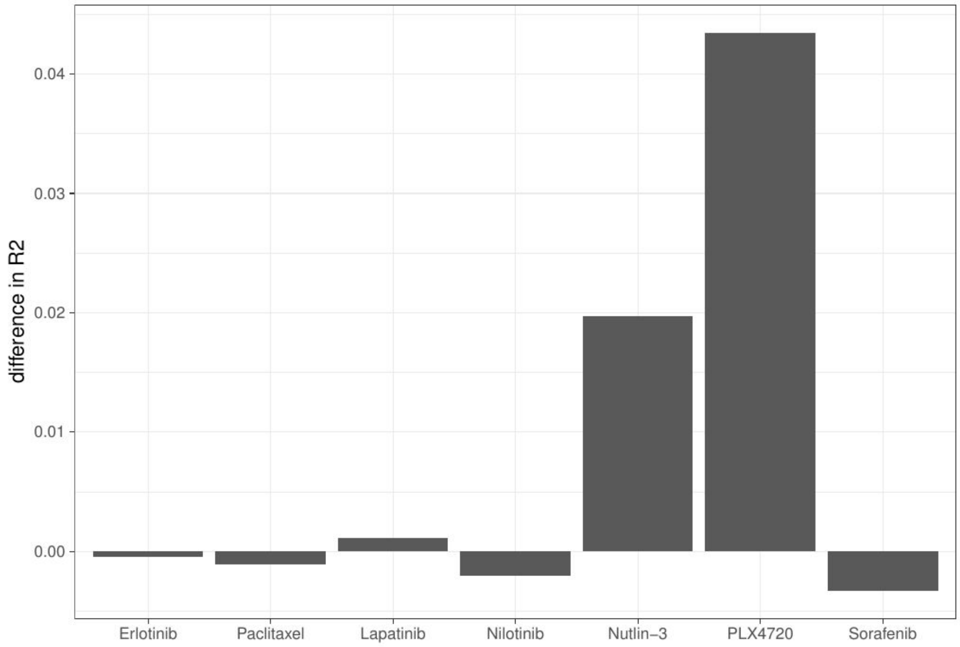

Figure s7. Difference in average R^2 between models that use all genomic features and models that use only expression features for 7 drugs.

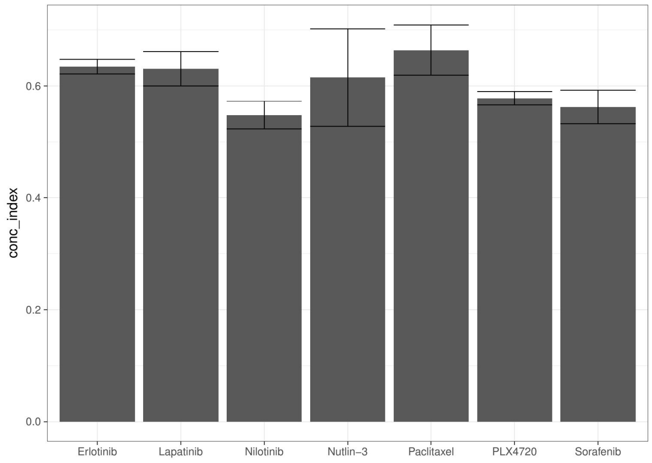

Figure s8. Average (across three datasets) concordance index values for each drug separately (for models with AUC metric).

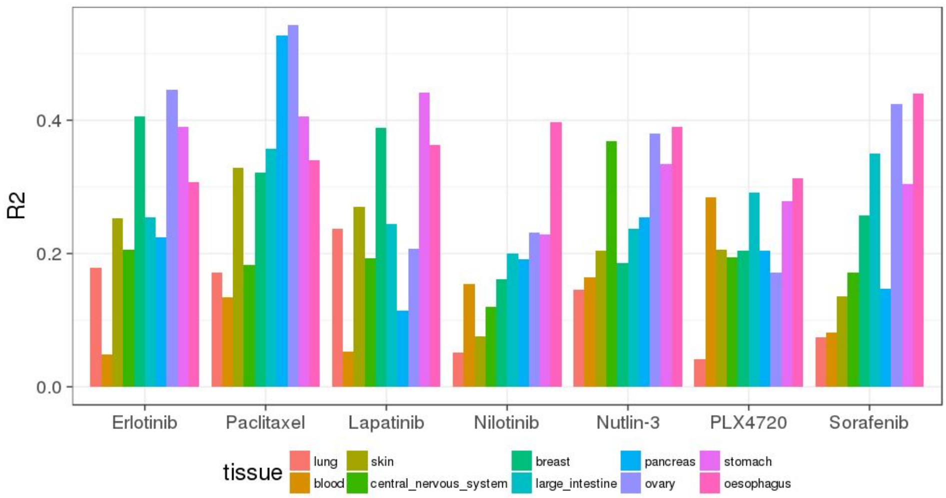

Figure s9. Average (across three datasets) R^2 values for each tissue and each drug separately (for models with AUC metric).

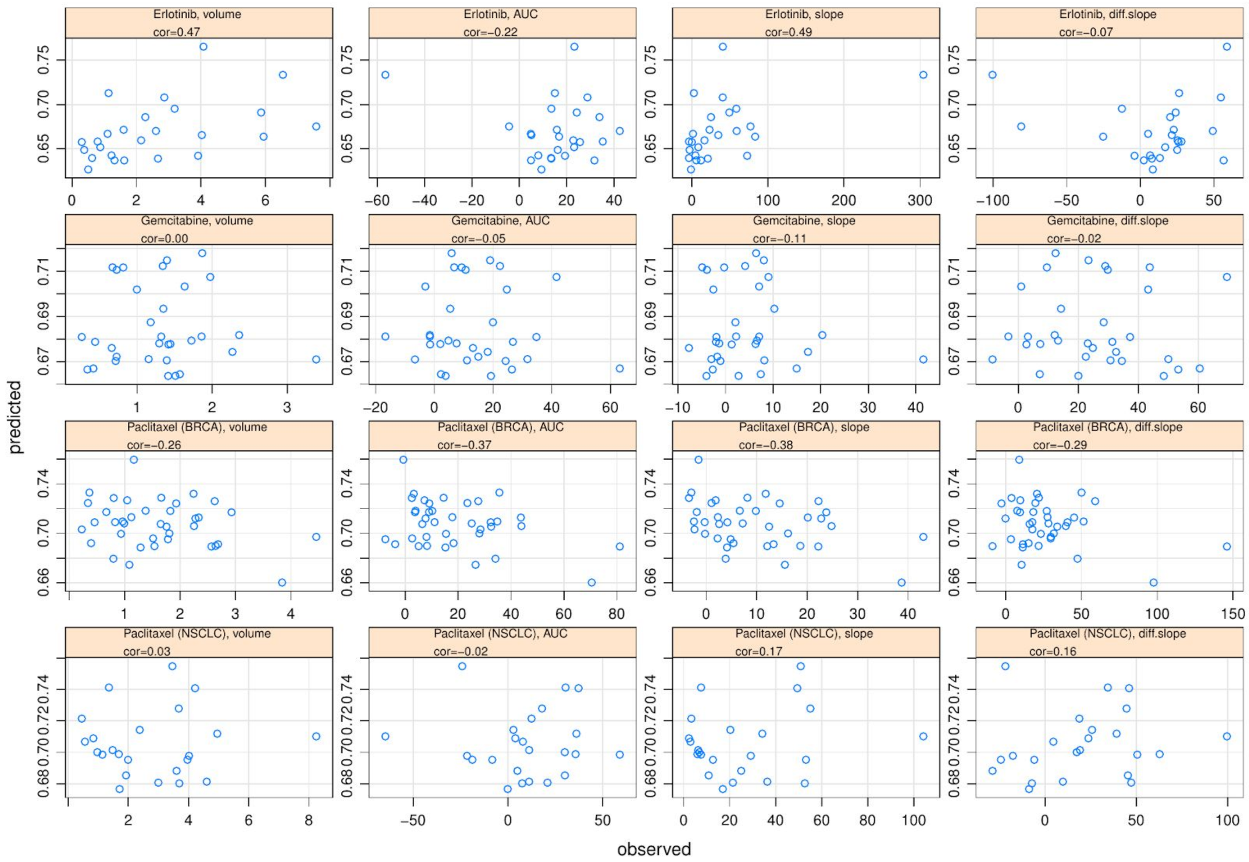

Figure s10. Observed vs. predicted values for [cell lines → xenografts] type of prediction and corresponding correlation coefficients

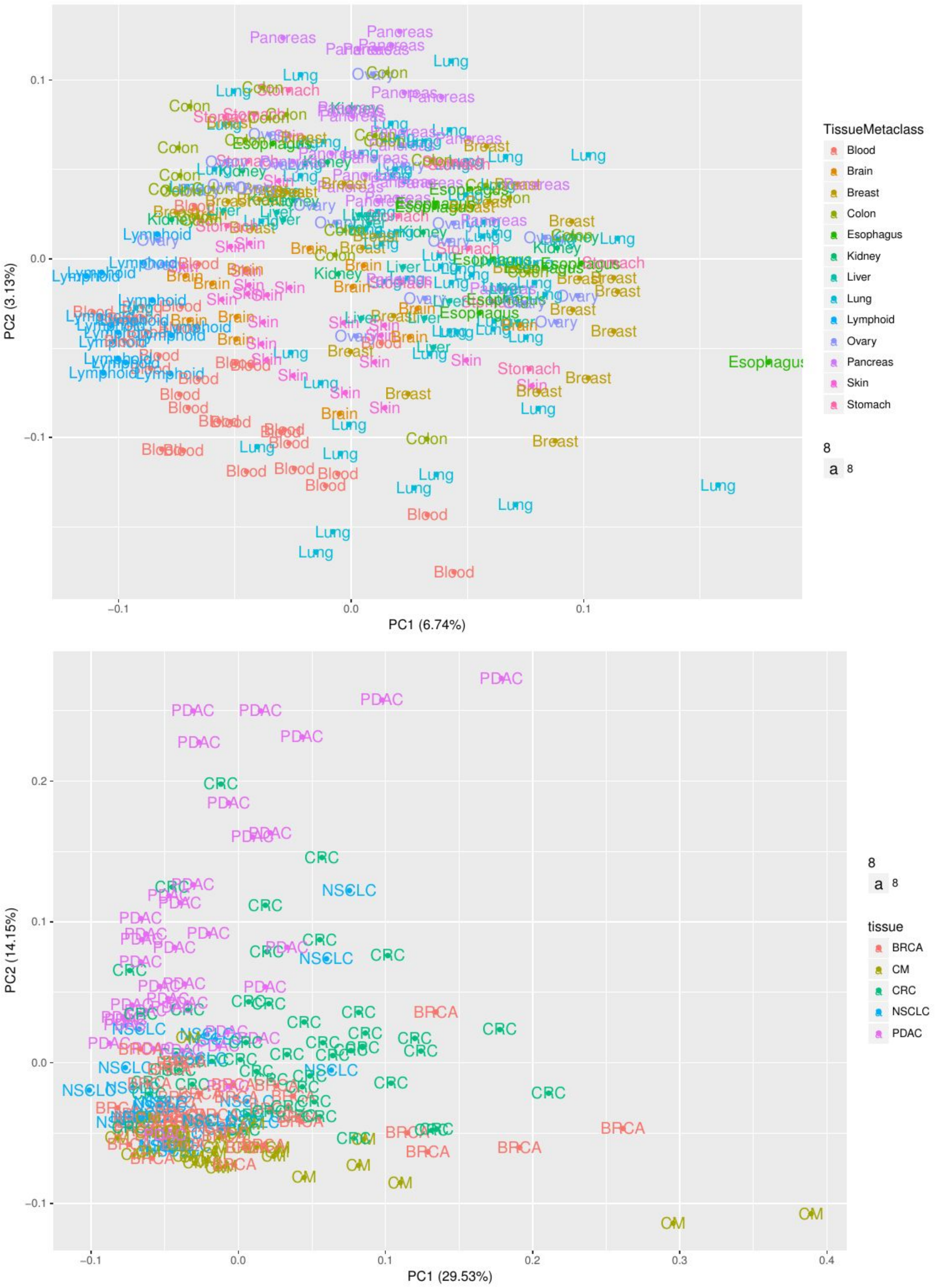

Figure s11. PCA plots based on molecular data with tissue labels for each samples. Top -- cell lines (gCSI), bottom -- xenografts (NIBR PDXE).

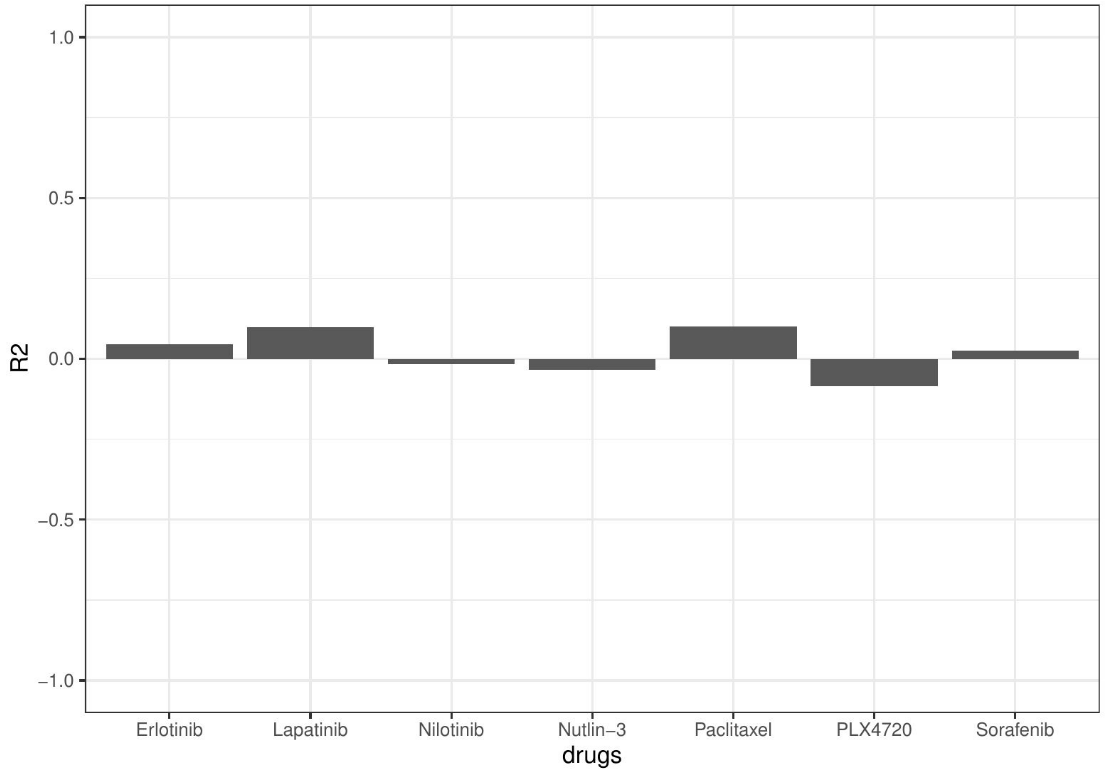

Figure s12. Average correlation between number of features in the model and resulting R2 for each drug. Plotted R2 are values averaged across datasets (CCLE, CTRP, GDSC) and drug response metrics (IC50, AUC, viability_1uM). In these tests we tested the following numbers of variables: 100, 500, 2500 and 5000.

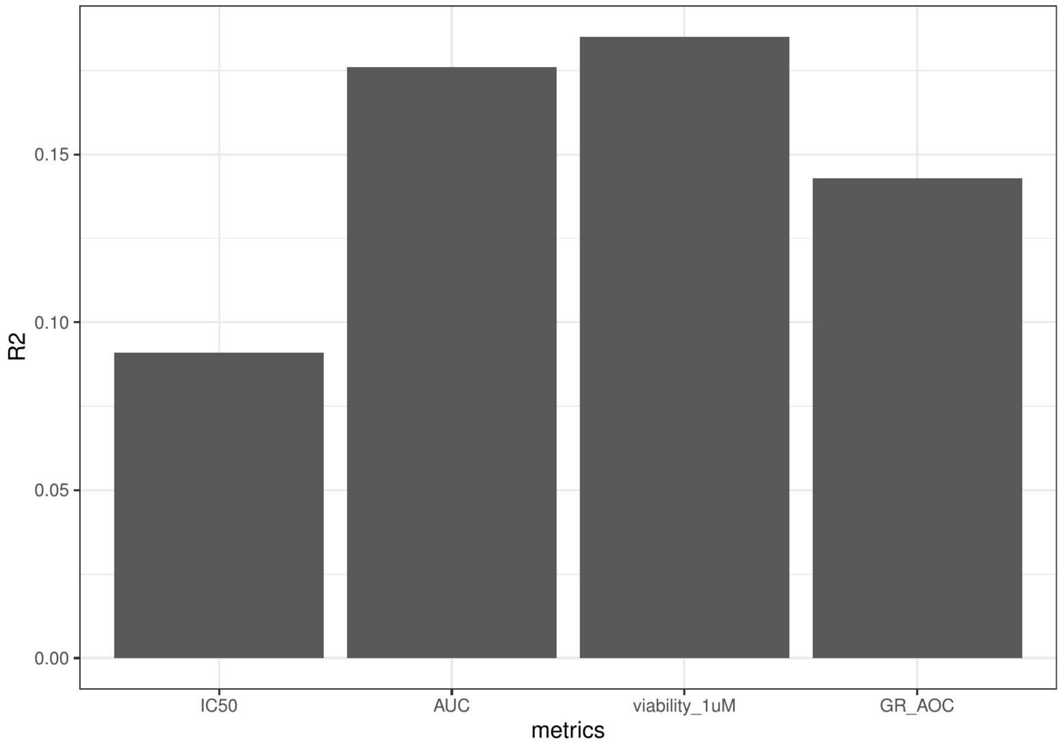

Figure s13. Average R2 across 7 drugs for different drug response metrics including GR_AOC metric. In these tests we used only data on 146 cell lines from CTRP dataset, since only on these cell lines we had GR_AOC values available via http://www.grcalculator.org.

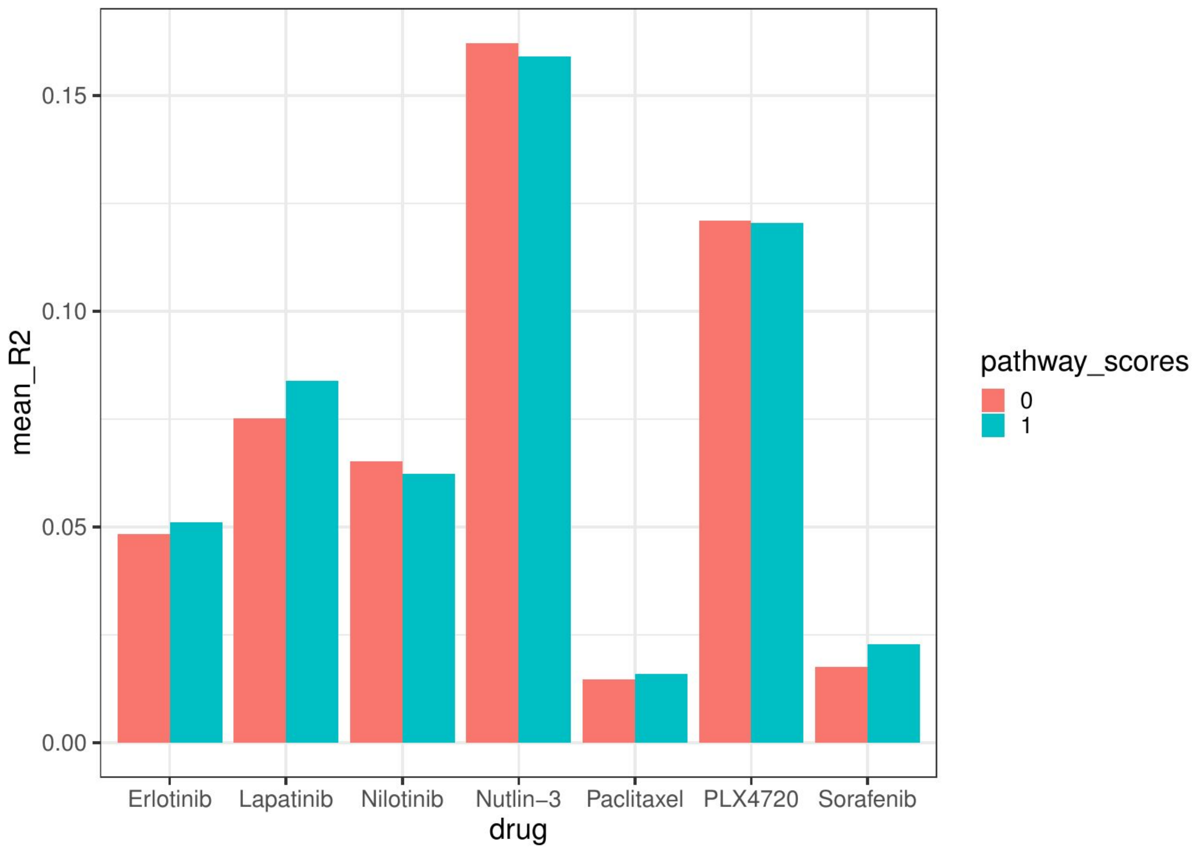

Figure s14. Average R2 across 7 drugs for models with (pathway_scores=1) and without (pathway_scores=0) pathway signature features calculated using PROGENy tool. Base model include 200 genomic features selected via feature selection procedure. For these tests we used only data from GDSC dataset.

	Overlap between top 400 features selected via anovaScores for the 2 classification tasks:<br>(lung vs. others[-breast]) and and (breast vs. others[-lung])		Overlap between gCSI and NIBR for the task<br>(breast+lung vs. others)
	gCSI	NIBR PDXE	
genes	APOBEC3C<br>C14orf162<br>C15orf59<br>CCDC149<br>CYP1B1<br>ENAH<br>FAM127C<br>FERMT2<br>FOXA1<br>HMGCLL1<br>LDOC1<br>LOC100507372<br>LOC115110<br>NOTCH3<br>NPR3<br>NRK<br>PALMD<br>SIX2<br>TNFRSF14<br>TRIM16L<br>ZNF793<br>ZSCAN18<br>ZYG11A	ARHGEF26-AS1<br>BCL6<br>BNIPL<br>CAMK1D<br>CD97<br>FBXO27<br>GRHL1<br>HMGB3<br>HS6ST1<br>IRX3<br>IRX4<br>KIAA0922<br>LTBP1<br>MTMR12<br>NOTCH3<br>NT5C3<br>NTN1<br>NXN<br>PACSIN3<br>PAPD7<br>PC<br>PGPEP1<br>PIAS3<br>PKP1<br>PLAC2<br>PSME4<br>PTPRF<br>PXDN<br>RGMA<br>RPS27L<br>SEMA4A<br>SIX4<br>SLC6A11<br>SLC6A9<br>SUSD4<br>TCF7L1<br>TMEM132A<br>TMEM25<br>TMPRSS13<br>UBE2E2<br>VIPR2<br>ZDHHC18<br>ZNF436<br>ZNF750<br>ZYG11A	ABHD14B<br>ARHGEF26-AS1<br>BNIP3<br>C10orf35<br>C5orf38<br>EFHD1<br>EFS<br>ENAH<br>FAM127C<br>GHDC<br>GIMAP2<br>GPR156<br>HMGB3<br>IRX2<br>IRX3<br>KIAA2022<br>LDOC1<br>LOC100506930<br>MB<br>NOTCH3<br>PALLD<br>PAQR8<br>PXDN<br>S1PR3<br>SNAP47<br>SUSD4<br>TMEM132A<br>TMEM25<br>TPBG<br>TSPYL5<br>VASN<br>ZNF512B<br>ZYG11A
DAVID clusters	DNA binding/transcription regulation/homeobox<br>Membrane/transmembrane		
		transcription from RNA polymerase II promoter<br>immunoglobulin domain<br>Zinc-finger<br>ATP-binding	leucine rich repeat

Table s1. Gene expression features, selected for different tissue type classification tasks, which can distinguish lung and breast samples from other samples. Top DAVID annotation clusters are also shown.

drug	Number of tested cell lines		
	CCLE	CTRP	GDSC
Erlotinib	494	764	323
Lapatinib	495	719	349
Nilotinib	410	753	646
Nutlin-3	493	751	662
Paclitaxel	492	708	357
PLX4720	486	760	662
Sorafenib	491	761	355

Table s2. Number of cell lines tested with each drug in CCLE, CTRP and GDSC datasets.

Drug, tissue	Number of tested samples	
	gCSI	NIBR PDXE
Erlotinib, lung	68	25
Gemcitabine, pancreas	26	32
Paclitaxel, breast	29	38
Paclitaxel, lung	68	23

Table s3. Number of cell line or xenograft samples from certain tissue tested with Erlotinib, Gemcitabine, and Paclitaxel in gCSI and NIBR PDXE datasets.
